# Identifying Chemicals with Potential Therapy of HIV Based on Protein-Protein and Protein-Chemical Interaction Network

**DOI:** 10.1371/journal.pone.0065207

**Published:** 2013-06-06

**Authors:** Bi-Qing Li, Bing Niu, Lei Chen, Ze-Jun Wei, Tao Huang, Min Jiang, Jing Lu, Ming-Yue Zheng, Xiang-Yin Kong, Yu-Dong Cai

**Affiliations:** 1 Institute of Systems Biology, Shanghai University, Shanghai, P. R. China; 2 Key Laboratory of Systems Biology, Shanghai Institutes for Biological Sciences, Chinese Academy of Sciences, Shanghai, P. R. China; 3 College of Life Science, Shanghai University, Shanghai, P. R. China; 4 College of Information Engineering, Shanghai Maritime University, Shanghai, P. R. China; 5 The Key Laboratory of Stem Cell Biology, Institute of Health Sciences, Shanghai Institutes for Biological Sciences, Chinese Academy of Sciences, Shanghai, P. R. China; 6 Department of Genetics and Genomic Sciences, Mount Sinai School of Medicine, New York, New York, United States of America; 7 Drug Discovery and Design Center (DDDC), Shanghai Institute of Materia Medica, Shanghai, P. R. China; Semmelweis University, Hungary

## Abstract

Acquired immune deficiency syndrome (AIDS) is a severe infectious disease that causes a large number of deaths every year. Traditional anti-AIDS drugs directly targeting the HIV-1 encoded enzymes including reverse transcriptase (RT), protease (PR) and integrase (IN) usually suffer from drug resistance after a period of treatment and serious side effects. In recent years, the emergence of numerous useful information of protein-protein interactions (PPI) in the HIV life cycle and related inhibitors makes PPI a new way for antiviral drug intervention. In this study, we identified 26 core human proteins involved in PPI between HIV-1 and host, that have great potential for HIV therapy. In addition, 280 chemicals that interact with three HIV drugs targeting human proteins can also interact with these 26 core proteins. All these indicate that our method as presented in this paper is quite promising. The method may become a useful tool, or at least plays a complementary role to the existing method, for identifying novel anti-HIV drugs.

## Introduction

Human immunodeficiency virus (HIV) is a lentivirus belonging to retrovirus family that causes acquired immunodeficiency syndrome (AIDS) [1], [2]. The global HIV and AIDS pandemic has caused nearly 60 million infections. Experts estimate that more than 25 million people have died of AIDS, and more than 33 million presently are living with HIV infection or AIDS [3].

During the last decade, the specific functions of HIV-1 encoded genes and related proteins have been extensively studied, which facilitated the development of the effective approved anti-AIDS drugs directly targeting the HIV-1 encoded enzymes, including reverse transcriptase (RT), protease (PR) and integrase (IN) [4], [5]. Despite the great efforts in developing new effective antiviral agents and the introduction of combination of these drugs, namely highly active antiretroviral therapy (HAART), the incidence of HIV infections continues to rise, because of the rapid emergence of drug-resistant HIV-1 mutants as well as the severe side effects. Therefore, there is an urgent need for further improvement of the existing anti-HIV drugs [6] and the introduction of novel drug design strategies [7] or novel antiviral targets with therapeutic potential for HIV infection [8].

Recently, it has been reported that several human proteins that were involved in HIV-1 life cycle and interactions with HIV-1 encoded proteins emerged as novel anti-HIV drug targets, including TSG101 [9], NF-κB [10], positive elongation factor P-TEFb [11] and cellular factors related to nuclear import of pre-integration complex [12]. Besides, small-molecule inhibition of the direct protein-protein interactions (PPI) that mediate numerous critical biological processes is an emerging area in current drug discovery [13], [14], [15], [16].

Multiple PPI involved in many biological processes in the HIV-1 life cycle have been identified by genomics, proteomics and biochemical approaches recently [17], [18], [19]. Although most of these interactions are complicated and have not yet been fully investigated, current knowledge on the molecular interactions has significantly broadened the understanding of the HIV-1 life cycle and paved an new way for the anti-HIV drug development. In fact, there is an increasing number of examples of both chemical and biological small molecular HIV inhibitors targeting PPI emerging nowadays [20].

In this study, we compiled all the PPI from HIV-1, Human Protein Interaction Database [17], [18], [19]. A PPI network was constructed with all these human proteins based on STRING [21] and 26 of them with a score greater than 1000 were selected according to their betweenness. Then, 280 chemicals in STITCH [22] that can interact with three HIV drugs targeting human protein were identified. It has been shown that these 280 chemicals can also interact with the 26 core human proteins. Therefore, the 280 chemicals and 26 human proteins may possess the potential for HIV therapy. Our method may open a new way for HIV drug design or at least plays a complementary role to the existing method.

## Materials and Methods

### HIV-1, Human Protein Interaction Data

All the protein-protein interactions (PPI) data were retrieved from the HIV-1, Human Protein Interaction Database (http://www.ncbi.nlm.nih.gov/RefSeq/HIVInteractions/) [17], [18], [19]. It includes 5,126 PPI and involves 19 HIV-1 proteins corresponding to 9 HIV-1 genes as well as 1,450 human proteins corresponding to 1,431 human genes. The PPI data was given in Additional File S1.

### Protein-Protein Interaction (PPI) Network

There are two PPI database: STRING (http://string-db.org/) [21] and HPRD (http://hprd.org/) [23]. The reasons why we chose STRING over HPRD are as following:

1). The STRING database includes more PPIs than HPRD. So far HPRD only contains 41,327 experiment supported PPI, while STRING contains 1,640,707 PPI including both direct ones (physical interactions) and indirect ones (functional interactions). HRPD is more likely to be a subset of STRING, since STRING includes the PPIs from experiments, existing databases, text-mining and predicted results.

2). The possible problems that the predicted PPIs with low confidence in STRING would cause can be avoided in our method. Since we used the weighted PPIs of STRING rather than the binary ones, the confidence of each PPI is considered. If a PPI has low confidence, it will be less important in Dijkstra’s algorithm during the shortest path analysis, and most likely to be eliminated.

3). The PPIs in HRPD is not weighted. Therefore, it is difficult to do quantitative network analysis. Overall, we selected STRING to construct the PPI network. Each interaction in STRING is evaluated by an interaction confidence score in range from 1 to 999 to quantify the likelihood that an interaction may occur. For clarity, let Q(p1,p2) denote the interaction confidence score of two proteins p1 and p2. The constructed network took proteins as its nodes, and the edge between any two nodes existed if and only if the corresponding proteins can interact with each other. To reflect the difference of interactions, each edge with endpoints v1 and v2 in the network was labeled with a score as the edge weight as follows:

 W(v1,v2) = 1,000−Q(p1, p2) (1)where p1 and p2 were corresponding proteins of nodes v1 and v2, respectively.

### Chemical-chemical Interactions and Protein-chemical Interactions

The data of chemical-chemical interactions and protein-chemical interactions was retrieved from STITCH (version 3.0) (http://stitch.embl.de/) [22], a well-known database containing 1,430,424 known or predicted chemical-chemical interactions between 89,617 chemicals as well as 1,221,559 protein-chemical interactions between 16,721 proteins and 234,826 chemicals deriving from experiments, literature or other reliable sources. Five scores with titles “Similarity”, “Experimental”, “Database”, “Textmining” and “Combined_score” in range from 1 to 999 were used to indicate the interactivity of two chemicals or a protein-chemical pair. Since the last score combines the information of others, it was used as the final interaction score.

### Shortest Path and Betweenness

For the given node in a network, its betweenness is related to the number of the shortest paths connecting all pair of nodes such that the node is the member of them [24]. For the node in PPI network, its betweenness accounts for direct and indirect influences of proteins at distant network [25]. Hence, betweenness has been used for study various natural and man-made networks [24], [26], [27], [28]. However, it is not necessary to calculate each node’s betweenness and consider all shortest paths. Here, we proposed a new kind of betweenness, named as betweenness related to A, where A was a node subset in a network. For this kind of betweenness, we only calculated the betweenness of the node in A not all nodes in the network. For a node d in A, its betweenness related to A, denoted by BA(d), was calculated by the following two steps: (1) Find shortest paths connecting all pair of nodes in A; (2) Count the number of the shortest paths such that d was the member of them.

## Results and Discussion

### 26 Core Human Proteins Identified According to their Betweenness

In our work, a protein-protein interaction network was constructed for the 1,450 HIV interacting proteins based on STRING. For each of the 1,450 proteins, its betweenness can be calculated according to the method in “Materials and methods”. In details, 1,050,525 shortest paths were found to calculate the betweenness related to 1,450 proteins. If a node appears in more than 0.1% of these shortest paths, it is deemed to be more important than other nodes. Thus we selected 26 proteins with betweenness greater than 1000, which were listed in Table 1. All the betweennesses for these 1,450 proteins were given in Additional File S2.

**Table 1:** 26 core human proteins identified by betweenness in shortest path.

Ensembl protein ID	Gene symbol	Betweenness
ENSP00000269305*	TP53	4611
ENSP00000263253*	EP300	4581
ENSP00000264657*	STAT3	3613
ENSP00000339007*	GRB2	2822
ENSP00000384273*	RELA	2762
ENSP00000226730*	IL2	2738
ENSP00000344818*	UBC	2518
ENSP00000360266*	JUN	2036
ENSP00000270202	AKT1	2032
ENSP00000275493	EGFR	1961
ENSP00000354394*	STAT1	1929
ENSP00000011653*	CD4	1796
ENSP00000229135*	IFNG	1434
ENSP00000353483*	MAPK8	1287
ENSP00000344456*	CTNNB1	1275
ENSP00000292303*	CCR5	1243
ENSP00000350941*	SRC	1161
ENSP00000341189*	PTK2	1151
ENSP00000348461*	RAC1	1147
ENSP00000329623*	BCL2	1123
ENSP00000329357*	SP1	1108
ENSP00000380227*	ITGA4	1076
ENSP00000226574*	NFKB1	1073
ENSP00000343204*	JAK1	1073
ENSP00000401303	SHC1	1063
ENSP00000228307	PXN	1053

* Directly interact with HIV proteins.

22 of these 26 proteins are well known to directly interact with HIV proteins in previous studies (Table 1). Their interactions include inhibition, activation, cleavage, degradation and so on (see Additional File S1), which should be deemed as causative. The rest four proteins may act as infection related, such as EGFR, which was upregulated by HIV-1 Gag protein. According to the roles played by these causative proteins during HIV life cycle, we briefly classified them into three groups, which respectively take part in receptor interaction, transaction and replication, and host immune response.

Within them, CD4 (cluster of differentiation 4) and CCR5 (C-C chemokine receptor type 5) are acting as co-receptors for HIV entry into targeting cells [29]. CD4 is a glycoprotein expressing on the surface of many kinds of immune cells such as macrophages, monocytes and T-help cells, and dendritic cells. It is recognized as the primary co-receptor of HIV targeting. It interacts with the viral envelope glycoprotein (Env) to trigger a structural alterations in Env and enable the virus to recruit other co-receptors, like CCR5 or CXCR4 [30]. The chemokine receptors CCR5, member of the seven-transmembrane G protein-coupled receptor superfamily, is one of the principal co-receptors for majority HIV isolates. It interacts with HIV protein gp120 so that HIV gp41 protein’s shape were changed to penetrate the cell membrane [31]. A natural mutant CCR5Δ32 (32 base pair deletion) can provide highly protection in HIV infected individuals in homozygous state [32], [33]. Besides, the small guanosine triphosphate hydrolase (GTPase) Rac1 (ras-related C3 botulinum toxin substrate 1) is reported to positively regulates co-receptor CXCR4 function [34].

The proteins mainly related to HIV transaction activity and replication in our result include TP53, EP300, STAT1, STAT3, GRB2, NF-κB complex subunit, polyubiquitin-C, Akt-1, interferon gamma, MAPK8, beta-catenin, SRC1, SP1, Bcl-2. Cellular tumor antigen p53 (TP53), a tumor suppressor participating in multiple pathway like cell cycle arrest or apoptosis, interacts with HIV-1 viral infectivity factor (Vif) to mediate G2 cell cycle arrest with a positive effect on HIV-1 replication [35]. Histone acetyltransferase p300 and SP1 interact with HIV-1 Viral protein-R (Vpr) to mediate Vpr activity in virion assemble, nucleus locating, promoter activation, cell cycle arrest or apoptosis induction [36]. In addition, Histone acetyltransferase p300, GRB2, Polyubiquitin-C, Akt-1, MAPK8 are all involved in the HIV trans- activating protein Tat mediated transactivation of HIV-1 LTR and viral replication, respectively [37], [38], [39], [40], [41]. Several proteins are to function by the similar pathway, such as the NF-κB signaling pathway or JAK-STAT pathway. Nuclear factor (NF)-κB complex is a master regulator of pro-inflammatory genes and is upregulated in HIV-1 infection. It plays a key role in the adaptive immune responses mounted against viruses, however, in addition to the protective effect, NF-κB may also contribute to viruses’ replication, survival and spread [42], [43], [44]. The JAK-STAT pathway usually transmits information from chemical signals outside the cell and involved in regulation of the immune system. Here, it includes Interferon gamma, STAT3, beta-catenin to regulate the HIV replication in astrocytes [45]. This also explains that the key proteins in these pathways ranking higher in our result.

The rest proteins are associated with the immune response against HIV infection. Interleukin-2 (IL-2), a secreted cytokine, is observed increasing in early CD4+ T-cell response for HIV-1 infection to control viral replication [46], though this response will lose function with the disease processing [47]. Transcription factor AP-1 is recruited by HIV Nef protein to MHC-I cytoplasmic tail to disrupt the presentation of HIV-1 epitopes to anti-HIV cytotoxic T lymphocytes [48]. The aberrant changes in pp125FAK expression block the beta1 integrin-mediated protection effect for aberrant cell death in patients with AIDS [49]. Integrin alpha-4 expressed on NK cells is bound by HIV gp120 to suppressing NK cells [50]. JAK-STAT pathway is also responsible for the antiretroviral effect of IFN-gamma, and the Jak/STAT deficiency may contribute to the dysfunction of CD4 T cell responses to a cytokine like IL-2 by HIV [51], [52].

### Chemicals Related to 26 Core Human Proteins

Three approved HIV drugs targeting human proteins in Drugbank were collected (Table 2).

**Table 2:** Three HIV drugs targeting human proteins in DrugBank.

Drugbank ID	Drug name	STITCH ID	Target gene
DB00900	Didanosine	CID100003043	PNP
DB00419	Miglustat	CID100051634	UGCG
DB04835	Maraviroc	CID100483407	CCR5

**Figure 1:**
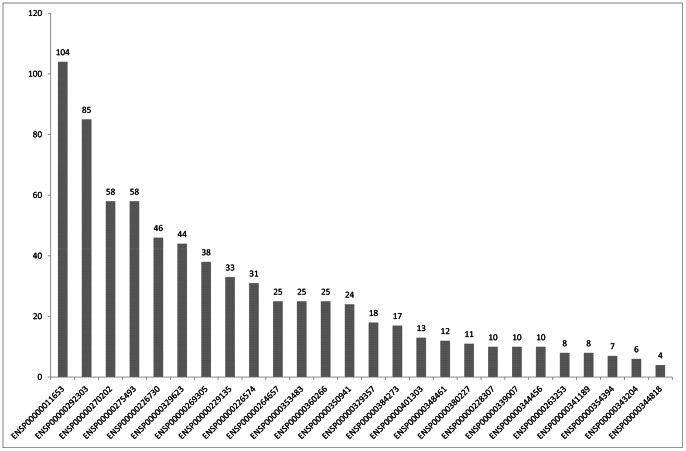
The number of interactive chemicals among 280 chemicals for each of the 26 core proteins.

For each of the three HIV related drugs, its interactive chemicals in STITCH can be found. After collecting these chemicals and combining with the three drugs, we obtained 280 chemicals (Additional File S3). For each of 26 proteins, we can count the number of the interactive chemicals among these 280 chemicals. Figure 1 shows the number of interactive chemicals among 280 chemicals. From Fig. 1, we can see that the number of chemicals related to ENSP00000011653 (CD4) is the largest, followed by that of ENSP00000292303 (CCR5). The chemicals related to each of the 26 proteins were ranked according to their interaction score (see Additional File S4).

### Chemicals Targeting Interaction between HIV and Human Co-receptors

**Figure 2:**
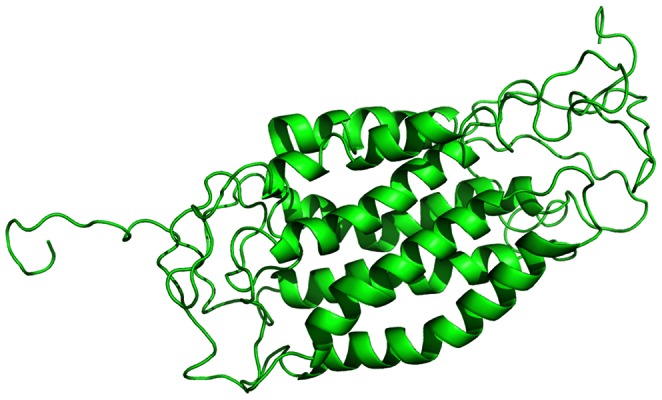
Three-dimensional structure of CCR5 based on PDB structure 1ND8 drawn with software Pymol. CCR5 is in green.

CCR5, a membrane protein, is an important target of anti-HIV therapy as it is one of the major co-receptors for HIV-1infection. There are seven trans-membrane helix structures in CCR5, which formed a “pocket” structure (Fig. 2) [53]. In this “pocket”, aromatic amino acid residuals, hydrophobic amino acid residuals, polar amino acid residuals and hydrophilicity amino acid residuals could bind with chemicals by π-π stacking interaction, hydrophobic interaction, hydrogen bonding interaction and salt-bridge interaction [54], [55], [56], [57].

**Figure 3:**
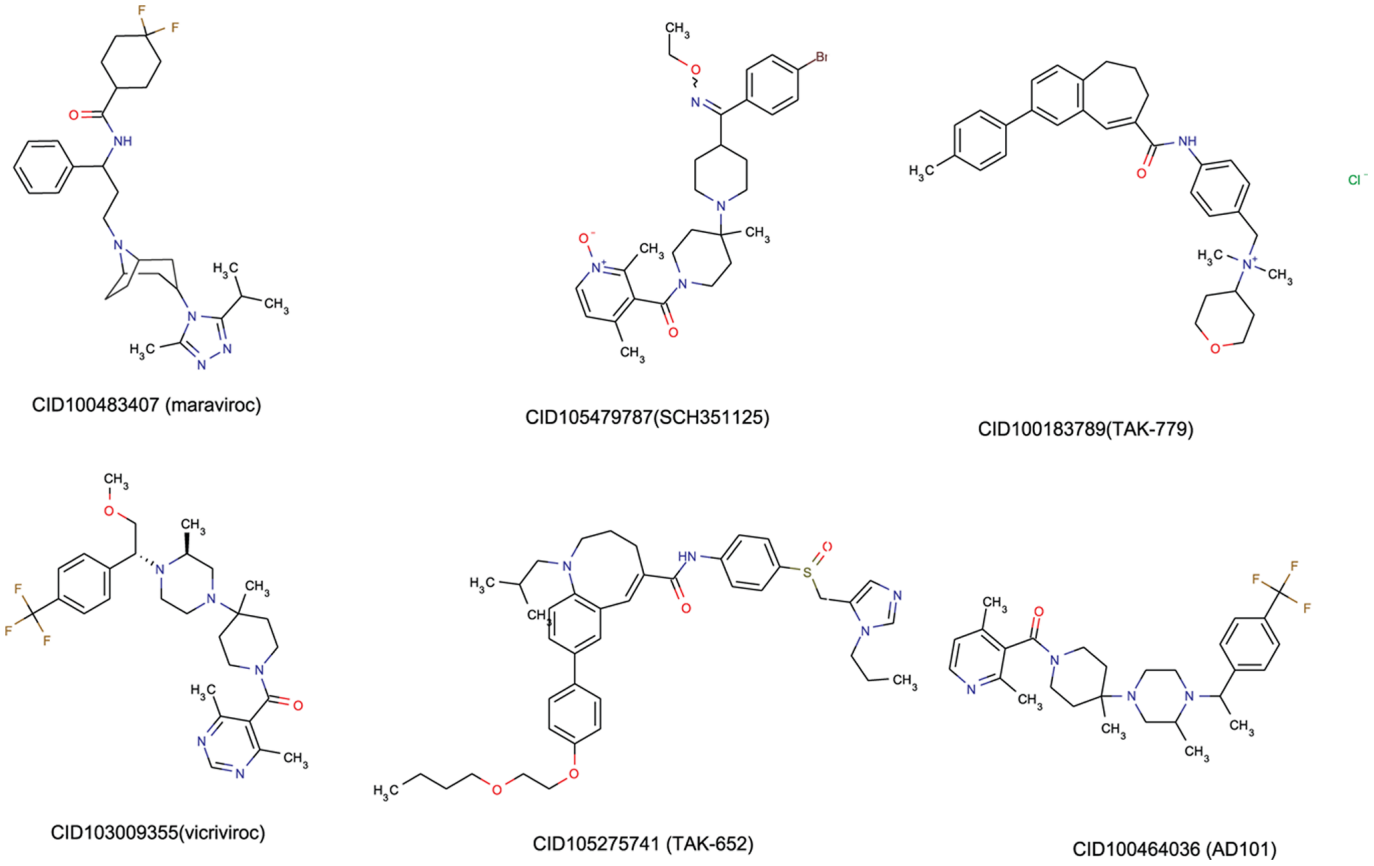
Structures of 6 chemicals whose interaction scores with ENSP00000292303 (CCR5) are greater than 900. The figure was generated using ChemAxon. The 6 chemicals are CID100483407 (maraviroc), CID105479787 (SCH 351125), CID100183789 (TAK-779), CID103009355 (vicriviroc), CID105275741 (TAK-652) and CID100464036 (AD101), which can also be found in PubChem with the IDs 483407, 5479787, 183789, 3009355, 5275741, and 464036, respectively.

For ENSP00000292303 (CCR5), it can be found that some chemicals with interaction score higher than 900 are very similar in sub-structure (Fig. 3). Among these chemicals, CID100483407 (maraviroc) (Table 2) is a known anti-HIV drug which could bind with CCR5. As for chemicals CID105479787 (SCH 351125), CID100183789 (TAK-779), CID103009355 (vicriviroc), CID105275741 (TAK-652), CID100464036 (AD101), they are very similar to CID100483407 (maraviroc) in sub-structure. We speculated these chemicals may also have the same target CCR5.

From Figure 3, it can be seen that all these chemicals have three hydrophobic structures containing basic nitrogen atoms (nitrogen atoms in bridge chain, piperidine ring, ammonium salt) and amide group. The three hydrophobic structures can interact with hydrophobic amino acid or hydrophobic structures of trans-membrane by hydrophobic interaction or π-π stacking interaction like CID100483407 (maraviroc). Benzene ring and Triazole ring of CID100483407 (maraviroc) can insert into hydrophobic pocket and form T shape π-π stacking by interacting with Tyrosine (Tyr108) and tryptophan (Trp86). In addition, cyclohexyl group of CID100483407 (maraviroc) can interact with isoleucine (Ile198) to form hydrophobic interaction. Furthermore, the interaction between basic nitrogen atoms in bridge chain of CID100483407 (maraviroc) and hydrophilicity amino acid residuals of CCR5 forms salt-bridge which is the major binding modes. Similar to CID100483407 (maraviroc), basic nitrogen atoms in bridge chain of CID105479787 (SCH 351125) can also form salt-bridge with glutamic acid (Glu283). Methylbenzene and formyl pyridine ring at nitrogen atom could form π-π stacking structure with tryptophan 86 and 248 (Trp86, Trp248), respectively. Meanwhile, acetyl group of piperidine ring and isoleucine (Ile198) could form hydrophobic structure. As for the other chemicals like CID100183789 (TAK-779), CID103009355 (vicriviroc), CID105275741 (TAK-652), CID100464036 (AD101), they are similar to CID100483407 (maraviroc). Therefore, they may be also considered as potential anti-HIV drugs targeting CCR5.

### Chemicals Targeting Interactions Involving HIV-1 Reverse Transcriptase

**Figure 4:**
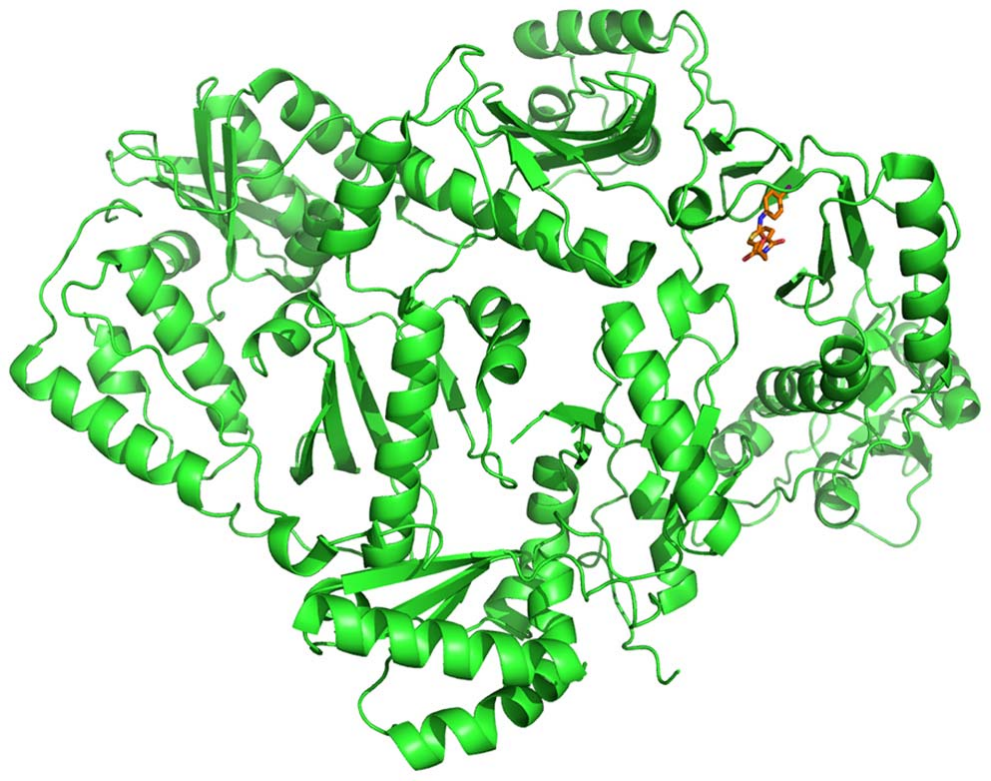
Three-dimensional structure of HIV-1 RT based on PDB structure 2VG7 drawn with software Pymol. HIV-1 RT is in green. Ligand is in orange, red and blue.

It can be found that some chemicals whose interaction score are higher than 740 for ENSP00000011653 (CD4) are also similar in sub-structure. Most of these chemicals are related to HIV-1 reverse transcriptase (HIV-1 RT) and HIV-1 Protease (HIV-1 PR). HIV-1 RT is a hetero-dimeric enzyme which is composed of two distinct subunits P66 and P51 [58], [59]. The peptide sequence of P51 is identical to the first 440 amino acids of P66, and they form the two subunits of polymerases domain. The subunit looks like human’s right hand which contains the finger, palm, thumb, and connection subdomains (see Fig. 4). The finger subdomain includes β-sheets and three α-helices, and the palm subdomain contains five α-helices. These α-helices and β-sheets of finger and palm subdomains could form hydrogen bonding structure with four β-sheets of thumb subdomain. The hand of the domain and the RNase H domain is connected by connection subdomain which is composed of a big β-sheet and two α-helices [58], [59], [60]. P66 also looks like a right hand, and it makes up a large template-primer binding cleft of polymerase. The 3′-OH terminus of the primer is positioned close to active site of polymerase (three catalytic amino acid residuals: Asp110, Asp185 and Asp186). P51 is processed by proteolytic cleavage of P66, which is different from P66 in structure although their amino acid sequences are similar [61]. The finger of P51 is close to the palm, and there is no template-primer binding cleft. As the active sites are buried, there is no catalytic activity for P51. Hence each P66/P51 dimer has only one active site which is located in P66. When HIV infects the host cell, HIV-1 RT creates single-stranded DNA from the RNA template. First, RT binds to RNA. Then the corresponding DNA nucleoside of host cell binds to phosphate group as substrate, and copy RNA nucleotide [61]. As RT is a essential enzyme during the replication of HIV-1, lack of HIV-1 RT could block the HIV-1 replication cycle, thus preventing HIV reproduction. Therefore, RT is regarded as an important anti-HIV target. At present, RT inhibitors could be classified to nucleoside analog reverse-transcriptase inhibitors (NARTIs) and non-nucleoside reverse-transcriptase inhibitors (NNRTIs).

**Figure 5:**
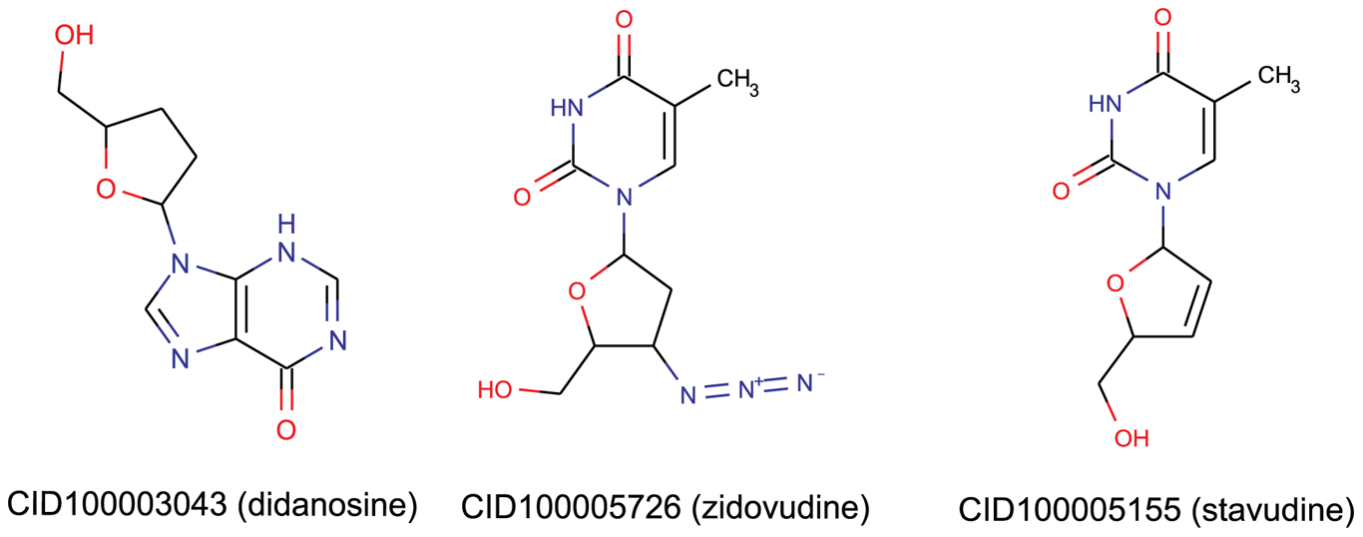
Structures for 3 chemicals whose interaction scores with ENSP00000011653 (CD4) are greater than 740. The figure was generated using ChemAxon. The 3 chemicals are CID100003043 (didanosine), CID100005726 (zidovudine) and CID100005155 (stavudine), which can also be found in PubChem with the IDs 3043, 5726 and 5155, respectively.

Among the chemicals with score greater than 740, CID100003043 (didanosine) (Table 2) is a known anti-HIV drug targeting HIV-1 RT. Intriguingly, we found that CID100005726 (zidovudine) and CID100005155 (stavudine) may also bind to HIV-1 RT. These three chemicals are nucleoside analog which are very similar to RNA and DNA in structure (Fig. 5). Nucleoside analogs could be phosphorylated when they enter the cells. Then they compete with natural deoxynucleotides for binding with RT, thus inhibit the usage of nucleoside substrates by RT, arrest the growing of viral DNA and prevent viruses’ reproduction [62], [63], [64]. In this study, CID100005726 (zidovudine), CID100005155 (stavudine), CID100003043 (didanosine) are phosphorylated to nucleoside 5′-monophosphate analog, nucleoside 5′-diphosphate analog, and nucleoside 5′-triphosphate analog, respectively, after the three chemicals enter the cells. Then the three analogs could bind with RT instead of natural nucleoside phosphate substrates (dTTP, dCTP, dATP, dGTP). As a result, the binding between natural nucleoside substrates and HIV-1 RT is blocked, and the HIV-1 RT is competitively inhibited. On the other hand, as there is no 3′-OH in these three chemicals, viral DNA could not grow after binding with the three chemicals. This could also prevent the HIV viruses’ reproduction.

**Figure 6:**
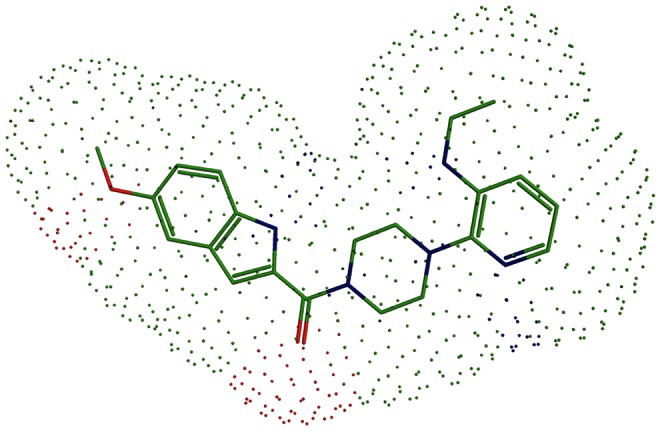
Non-nucleoside analog’s butterfly-like shape drawn with software chemoffice and Pymol. The structure was retrieved from PubChem with the ID 60847. Dot surface is colored according to atom types.

Other chemicals targeting HIV-1 RT including CID100060847 (BHAP), CID100004463 (nevirapine), CID105495818 (BMS-378806) could be classified to NNRTIs. Different to NARTIs, NNRTIs have two symmetrical aromatic rings, which show special butterfly-like shape (Fig. 6) [65], [66]. Five β-sheets of P66 form a pocket which is composed of hydrophobic amino acids. Based on hydrophobic, hydrogen bonding and π-π stacking interaction derived from aromatic ring of aromatic amino acids, the aromatic rings from one side of these three chemicals can interact with aromatic amino acids including Tyr181, Tyr188, Phe227, Trp229 and those from another side can interact with hydrophobic amino acids including Val179, Val106 and Leul00. Therefore, a small hydrophobic pocket is formed by Tyr181, Tyr183 and Tyr188. As the three amino acids rotate outside, the entrance of the pocket will be exposed where HIV-1 RT can bind to these three chemicals. In this case, the relative locations of β4, β7 and β8 sheets will change. Complementary rearrangement of the conformation of RT and CID100060847 (BHAP), CID100004463 (nevirapine), CID105495818 (BMS-378806) result in hydrophobic interactions [64], [67], [68]. As a result, the conformation of the newly located catalytic active site is similar to that of P51 [16]. Therefore, the new conformation is inactive. This is the reason why non-nucleoside analog has the ability to inhibit the RT by changing the conformation of catalytic site.

### Chemicals Targeting Interactions Involving HIV-1 Protease

**Figure 7:**
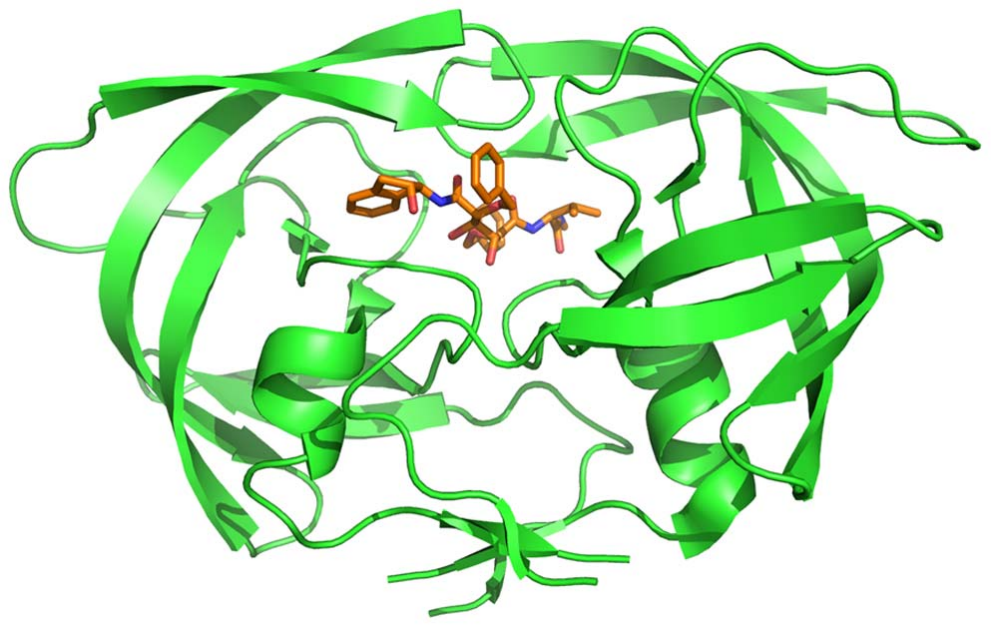
Three-dimensional structure of HIV-1 PR based on PDB structure 1EBZ drawn with software Pymol. HIV-1 PR is in green. Ligand is in orange, red and blue.

HIV-1 protease is a C2-symmetric homodimer including two monomers which have the identical polypeptide sequence with 99 residues (see Fig. 7) [69]. There is an active site (Asp-Thr-Gly) in the region between P25 and P27. The two subunits are connected by four β anti-parallel strands containing glycine, and each strand contains N-terminal domain and C-terminal domain. Both the monomers have a long “cavity” structure, on the bottom of which lie the catalytic aspartyl residues with planar configuration [69], [70]. Due to the special structure of HIV-1 PR, the substrate peptide binds to the enzyme in an extended anti-parallel β sheet through the amino acid side chains from completely opposite directions [71]. It should be noted that the two subunits of enzyme are not completely identical, although they are symmetrical. Both the monomers have a “flap” structure which is made up of antiparallel β strands extending to subsite P1 and P2’ [72]. Due to the different conformation of ‘flap’ of the two subunits, such a symmetrical conformation has the ability to recognize particular amino acid residues to control the substrates/inhibitors’ access.

Both CID100003706 (indinavir) and CID100005076 (ritonavir) are peptidic chemicals which could compete with natural substrates as the substrates of HIV-1 PR. During the process of hydrolysis of peptide bonds of these two chemicals, one active water molecule is polarized by carboxyl group of Aspartyl residue. Nucleophilic O-atom of water attacks the carbonyl of the substrate’s scissile bond to form a tetrahedral intermediate which could further become amino and carbonyl chemical. Then the hydroxy and carboxyl group form hydrogen interacts with Aspartate as hydrogen bond acceptor and donor, respectively. Meanwhile, aromatic rings of side chain of these two chemicals firmly bond to active region of protease through electrostatic and steric interactions. As a result, the conformation of “flap” changes and a tunnel structure which go through the dimmer obliquely forms in the active region. Then the symmetrical conformation of protease is broken and the flexible region closes. Eventually, HIV-1 PR is inhibited by damaging its activity.

### Miglustat may also Target HIV-1 Protease

Miglustat is another approved drug for HIV in Drugbank with the identity of CID100051634 (Table 2). It was revealed by our result that CID100051634 is also related to ENSP00000011653 (CD4), though interaction score of CID100051634 is only 359. However, it can be found that the structure of CID100051634 is still similar to the sub-structure of peptidic chemical. And the related studies show that CID100051634 also have anti-HIV activity in experiment. CID100051634 is an N-alkylated imino sugar. Clinical trials show that CID100051634 alter the glycosylation of envelope glycoproteins and decrease the infectivity in certain viral diseases such as HIV [73]. Carefully study CID100051634, we can found that CID100051634 has three hydroxyl groups which can interacts with Aspartate as hydrogen bond acceptor and donor, respectively. Therefore, we presume CID100051634 may also target HIV-1 PR.

### New Combinations of HAART Proposed by Chemical-chemical Interaction

A combination of HAART generally includes two NARTIs and one drug in the following classes: NNRTI, protease inhibitor (PI), integrase strand transfer inhibitor (INSTI), or a CCR5 antagonist. According to the presumption that “two interactive chemicals are more likely to share similar biological functions [74], [75], [76]”, we attempted to propose some new combinations of HAART through substituting the original components for their interactive chemicals. One way is replaced by inhibitors in the same class. NIH proposed some preferred regimens of HAART with optimal, durable efficacy, favorable tolerability and toxicity profile [77], such as atazanavir/ritonavir+tenofovir disoproxil fumarate/emtricitabine (ATV/r+TDF/FTC). TDF/FTC is often used as a backbone for boosted PI-based regimens in the initial treatment of HIV-1 infection [78]. Therefore, we attempted to substitute ATV for other PIs. We found 166 interactive chemicals of atazanavir from STITCH [22], [79]. Except sulfate, eight interactive chemicals with the highest confidence score are PIs, including lopinavir (LPV), darunavir (DRV), ritonavir (RTV), saquinavir (SQV), fosamprenavir (FPV), nelfinavir (NFV), amprenavir (APV), and indinavir (IDV). All of these eight interactive chemicals related to ENSP00000292303 (CCR5), which is also shared by ATV. The combinations of one of the first five PIs with TDF/FTC are recognized regimens for anti-HIV-1 therapeutics by NIH [77]. NFV+TDF/FTC is also used for clinical AIDS treatment, although this medication may cause life-threatening lactic acidosis [80]. APV combined with TDF/FTC have been observed to have additive synergistic effects for antiretroviral therapy [81]. Thus, it is reasonable to assume the validity of IDV+TDF/FTC for the treatment of AIDS, but it need the safety assessment.

Another way is substituted for inhibitor in different classes. For example, Trizivir (abacavir+lamivudine+zidovudine, ABC+3TC+AZT) is recommended as an initial antiretroviral therapy [77]. Here we took 3TC+AZT as a backbone, and substituted ABC for inhibitors in different classes. Three inhibitors with the highest interaction score, efavirenz (EFV), nevirapine (NVP) and delavirdine (DLV), are NNRTIs. ABC and its three interactive chemicals are associated with ENSP00000011653 (CD4) and ENSP00000292303 (CCR5). EFV or NVP with 3TC+AZT are recognized by NIH [77], so we thought it was feasible to use DLV+3TC+AZT for antiretroviral therapy.

### Conclusion

At present, there is a great need for alternative way of inhibition for the design of anti-HIV therapeutics, because of the increased resistance of HIV to already approved drugs. Recently, inhibition of protein-protein interactions in the HIV life cycle is increasingly recognized as a valuable new avenue in drug design. In this work, we identified 26 core human proteins which play important roles in the HIV life cycles by interacting with HIV encoded proteins. In addition, 280 chemicals that interact with three HIV drugs targeting human proteins can also interact with these 26 core proteins. Therefore, the 280 chemicals may possess the potential for HIV therapy through intervention of PPI between 26 core human proteins and HIV encoded proteins. Our method may open a new way for HIV drug design or at least plays a complementary role to the existing method.

## Supporting Information

Additional File S1 **Protein-protein interaction information between HIV and human.**
(XLS)

Additional File S2 **All the betweennesses for the 1450 proteins involved in interaction with HIV encoded proteins.**
(XLSX)

Additional File S3 **The information of 280 chemicals.**
(XLSX)

Additional File S4 **The interacting chemicals for each of the 26 core proteins in 280 chemicals.**
(XLSX)
